# A new polychelidan lobster preserved with its eggs in a 165 Ma nodule

**DOI:** 10.1038/s41598-020-60282-1

**Published:** 2020-02-27

**Authors:** Clément Jauvion, Denis Audo, Sylvain Bernard, Jean Vannier, Allison C. Daley, Sylvain Charbonnier

**Affiliations:** 1 Muséum national d’Histoire naturelle, Sorbonne Université, CNRS UMR 7207, CR2P, Centre de Recherche en Paléontologie - Paris, 8 rue Buffon, 75005 Paris, France; 2 Muséum national d’Histoire naturelle, Sorbonne Université, CNRS UMR 7590, IRD, Institut de Minéralogie, de Physique des Matériaux et de Cosmochimie, IMPMC, Paris, France; 3 Yunnan Key Laboratory for Palaeobiology, Yunnan University, Kunming, China; 4 MEC International Joint Laboratory for Palaeobiology and Palaeoenvironment, Yunnan University, Kunming, China; 5 Univ Lyon, Université Claude Bernard Lyon 1, ENS de Lyon, CNRS, UMR 5276 LGL-TPE, 2, rue Raphaël Dubois, 69622 Villeurbanne Cedex, France; 6 Institute of Earth Sciences, University of Lausanne, Géopolis, CH-1015 Lausanne, Switzerland

**Keywords:** Palaeontology, Palaeontology

## Abstract

Crustacean eggs are rare in the fossil record. Here we report the exquisite preservation of a fossil polychelidan embedded within an unbroken nodule from the Middle Jurassic La Voulte-sur-Rhône Lagerstätte (France) and found with hundreds of eggs attached to the pleon. This specimen belongs to a new species, *Palaeopolycheles nantosueltae* sp. nov. and offers unique clues to discuss the evolution of brooding behaviour in polychelidan lobsters. In contrast to their development, which now relies on a long-lived planktic larval stage that probably did not exist in the early evolutionary steps of the group, the brood size of polychelidan lobsters seems to have remained unchanged and comparatively small since the Jurassic. This finding is at odds with reproductive strategies in other lobster groups, in which a long-lived planktic larval stage is associated with a large brood size.

## Introduction

Pleocyemata (including crabs, lobsters, crayfishes, caridean shrimps, polychelidan lobsters and others) is a group of decapod crustaceans characterised by a specific reproductive strategy, in which females brood their eggs on their abdominal appendages, instead of releasing them directly into the ocean.

**Figure 1:**
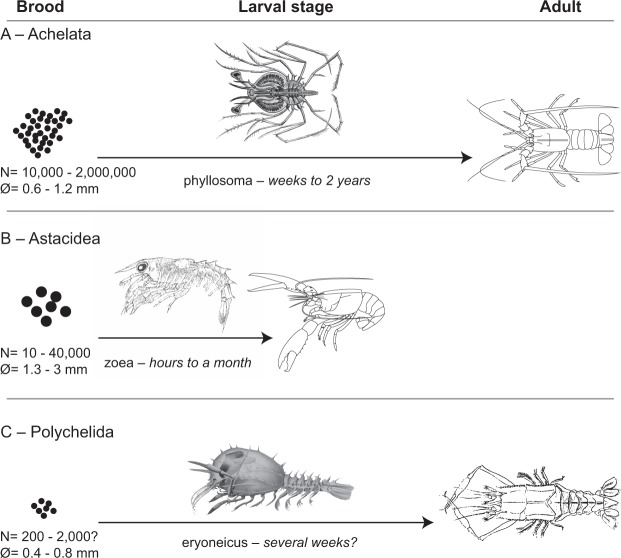
Comparative brood and development strategies in some extant lobsters (Decapoda). (**A**) Achelata Scholtz & Richer, 1995, eggs, phyllosoma (by C. Jauvion after Haeckel 1899), adult (by D. Audo); (**B**) Astacidea Latreille, 1802, eggs, third zoea (by C. Jauvion after Herrick 1911), adult (by D. Audo); (**C**) Polychelida Scholtz & Richer, 1995, eggs, eryoneicus (by C. Jauvion after Bouvier 1917), adult (by C. Jauvion after Hickson 1893).

**Figure 2:**
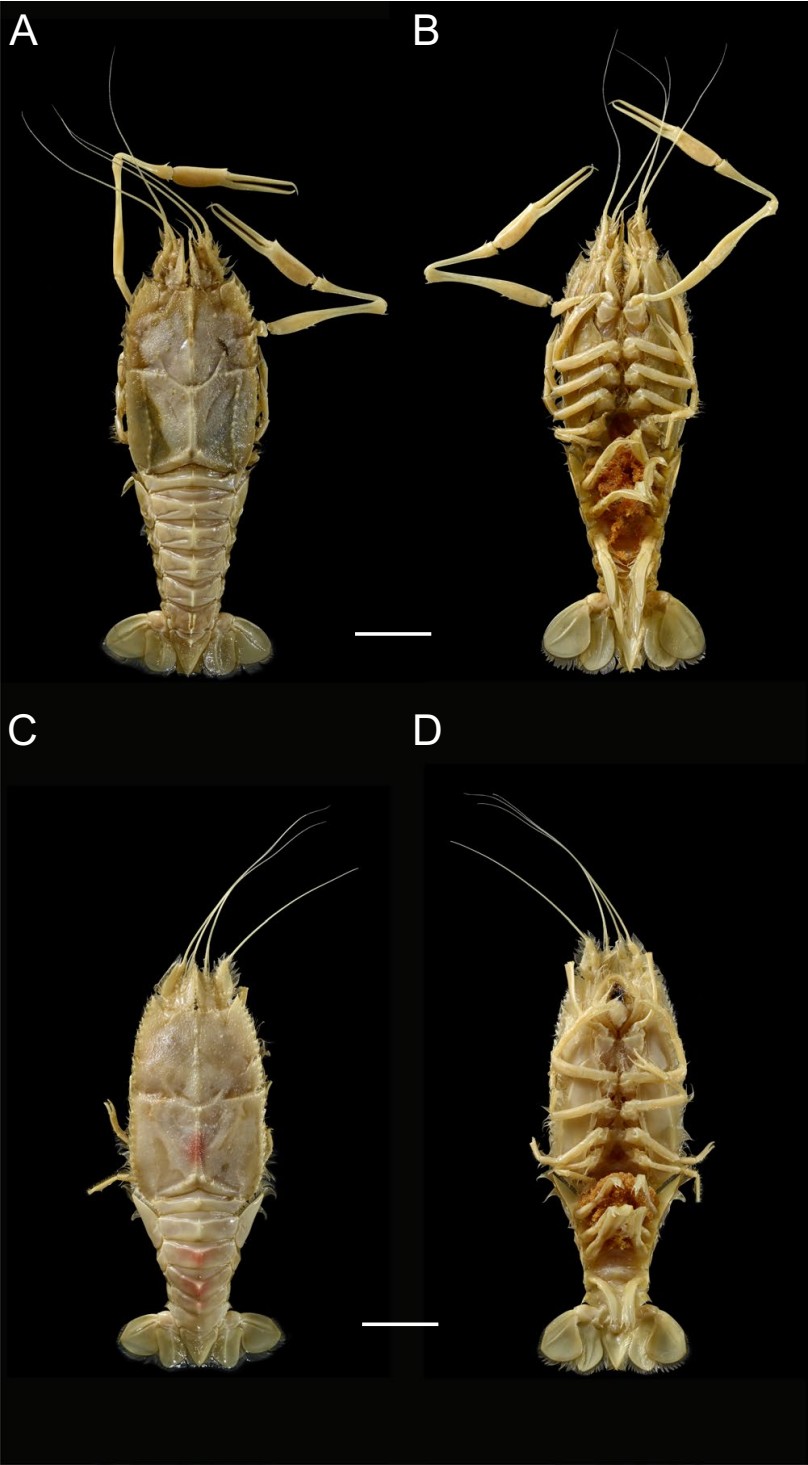
Modern ovigerous polychelidan lobsters; (**A**,**B**) *Stereomastis auriculata* (Bate, 1878), MNHN-IU-2016-9911; (**A**) dorsal view; (**B**) ventral view, 553 distinguishable eggs in this photograph; (**C**,**D**) *Polycheles enthrix* (Bate, 1878), MNHN-IU-2018-4209: (**C**) dorsal view; (**D**) ventral view, 389 distinguishable eggs in this photograph. Scale bars: 20 mm. Photographs: L. Cazes.

The development of modern polychelidan lobsters (Polychelida), a group of deep-sea dwelling Pleocyemata^1–4^ involves a long-lived, giant, balloon-like larval stage, the eryoneicus, which feeds within the plankton. In contrast to other Pleocyemata with a long-lived larval stage^5^, polychelidan lobsters have a low number (hundreds) of small eggs (Figs. 1 and 2). Although polychelidan lobsters have a long fossil record dating back to at least the Triassic^6^, we lack key information on the evolution of their reproductive modes. There is no evidence of eryoneicus larvae in the fossil record probably until at least the Late Cretaceous^7^. In contrast, there are multiple reports of immature adult-looking specimens from the Jurassic^3,8,9^ (Fig. S1), suggesting that the development of polychelidan lobsters did not rely on a long-lived planktic stage in Jurassic waters.

Although a few fossils of arthropods with preserved egg clutches have been described^10–17^, no fossil of a decapod crustacean with its eggs preserved has been reported so far, despite the discovery of several tens of well-preserved fossil specimens^18^. As a result, the number and size of eggs of Jurassic polychelidan lobsters was unknown, making it difficult to evaluate if the emergence of the eryoneicus larvae was accompanied by a change in fecundity.

**Figure 3:**
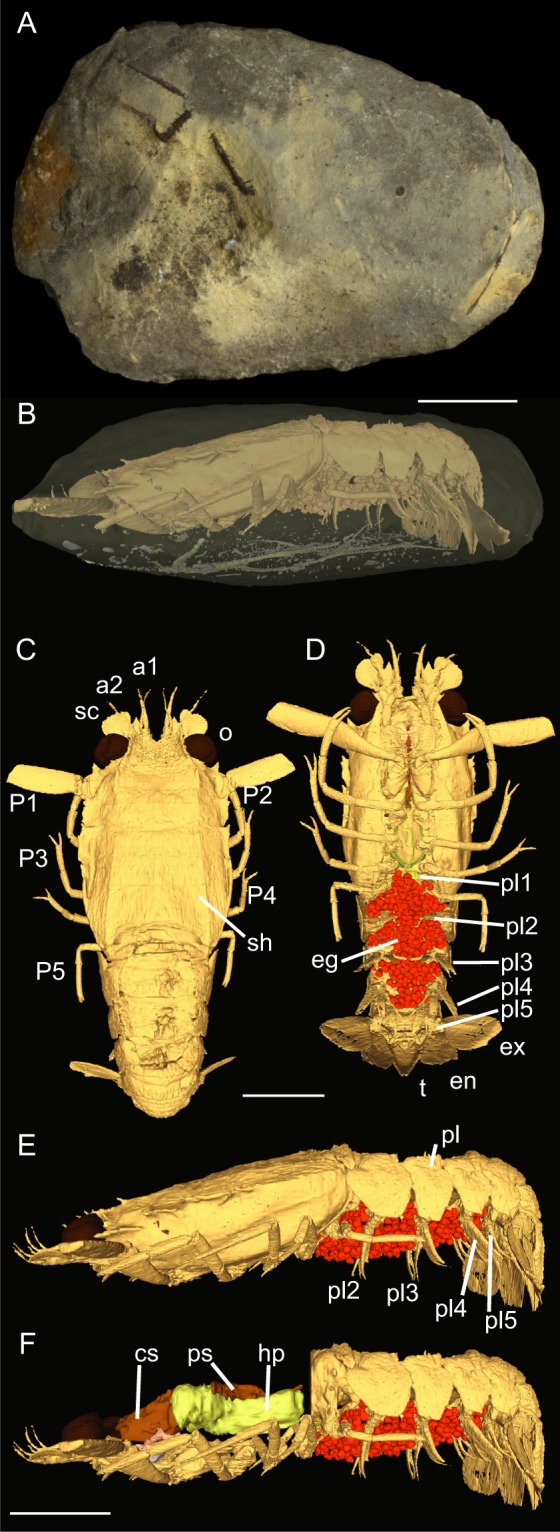
*Palaeopolycheles nantosueltae*, MNHN.F.A58254; (**A**) unbroken nodule showing arms of brittle stars; (**B–F**) 3D model; (**B**) nodule with the specimen and associated brittle stars visible; (**C**) dorsal view; (**D**) ventral view; (**E**) left lateral view; (**F**) left lateral view, with the shield removed. a1, antennula; a2, antenna; cs, cardiac stomach; eg, eggs; en, uropodal endopod; ex, uropodal exopod; hp, hepatopancreas; o, eye; P1-5, pereiopods 1–5 (thoracopods 4–8); pl, pleon; pl1-5, pleopods 1–5; ps, pyloric stomach; sc, scaphocerite; sh, shield; t, telson. Scale bars: 10 mm. Photograph: P. Massicard.

Here, we report the discovery of a unique specimen of polychelidan exquisitely preserved with its eggs within an unbroken nodule from the Middle Jurassic La Voulte-sur-Rhône Lagerstätte, France (Fig. 3).

## Results

**Figure 4:**
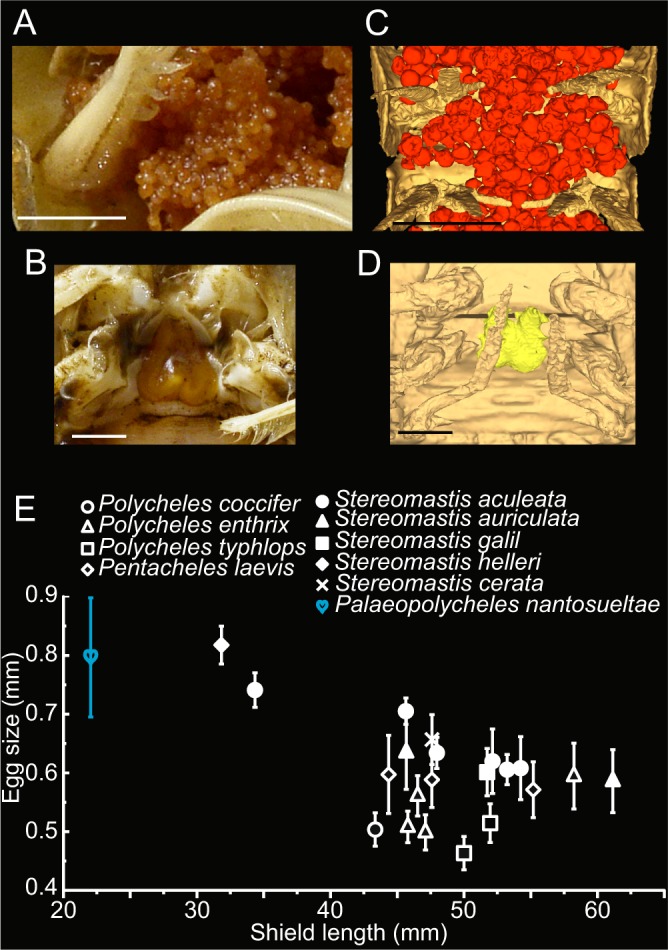
Modern eggs and spermatheca from polychelidans (**A**,**B**) and comparison with Jurassic *Palaeopolycheles nantosueltae* (**C**,**D**); (**A**) modern polychelidan eggs, *Stereomastis galil* Ahyong & Brown, 2002, MNHN-IU-2016.991; (**B**) modern spermatheca, *Polycheles coccifer* Galil, 2000, MNHN-IU-2018–4214; (**C**) close-up of preserved eggs; (**D**) close-up of preserved spermatheca; (**E**) Egg size and shield length of modern Polychelida and of *Palaeopolycheles nantosueltae*. 10 eggs were measured per specimen, error bars represent standard deviation. The egg size is not statistically different from that of MNHN-IU-2008-10470 (*Stereomastis helleri*; Wilcoxon-Mann-Whitney test, U = 38, p = 0.38396, N = 10). Scale bars: 5 mm in A and C; 2 mm in B and D. Photographs: L. Cazes.

X-Ray microtomography allowed the discovery and detailed reconstruction of anatomical features without damaging the sample. This is the sole representative of a new species. The investigated specimen displays a low number (n = 459) of relatively small-sized eggs under its pleon (abdomen), attached to the pleopods (swimming legs; Figs. 3 and S2). In addition to its eggs preserved in biological position, this specimen also displays a spermatheca, a specific organ used for sperm storage (Fig. 4D) present in modern Decapoda. All the mouthparts, the hepatopancreas (digestive gland), and the digestive tube (e.g. cardiac and pyloric stomachs; Fig. 3) are also preserved.

The position of eggs (under the pleon, carried by pleopods 1–5), their number (n = 459) and the size range (0.7–0.9 mm) are remarkably similar to those of modern polychelidan lobsters (Figs. 4 and S1). These clues suggest that Jurassic and extant polychelidan lobsters had a comparable reproduction mode and a relatively small brood size compared with other Pleocyemata. For comparative purposes, a new database of egg sizes in modern polychelidan lobsters was generated (Table S2).

### Systematic palaeontology

Eucrustacea Kingsley, 1894

Decapoda Latreille, 1802

Pleocyemata Burkenroad, 1963

Polychelida Scholtz & Richter, 1995

*Palaeopolycheles* Knebel, 1907

Type species. – *Eryon longipes* Fraas, 1855, by monotypy (Late Jurassic, Kimmeridgian, Germany).

*Palaeopolycheles nantosueltae* sp. nov.

Type material. – Holotype by monotypy (MNHN.F.A58254) from the La Voulte-sur-Rhône Lagerstätte, France (Middle Jurassic, Callovian^19^). Only known specimen of this species.

Etymology. – The specific epithet is referring to Nantosuelta, a Celtic goddess associated with fertility, alluding to the ovigerous state of the holotype.

*Palaeopolycheles nantosueltae* is ascribed to Polychelida based on the presence of chelate pereiopods 2–4, a concave frontal margin, and a dorsoventrally flattened body (Fig. S3). More precisely, it can be ascribed to *Palaeopolycheles* by its long anterolateral angle forming a spine orientated forward, ocular incision opening laterally, small cervical and hepatic incisions, short posterolateral angle, pleonite 5 on which the posterior transverse groove intersects the median line, very rounded scaphocerite, and narrow third maxilliped ischium^20^. *Palaeopolycheles nantosueltae* differs from the only other known species, *Palaeopolycheles longipes* (Fraas, 1855), by its shorter antennular peduncle reaching less than half the length of the scaphocerite (almost as long as the scaphocerite in *P. longipes*) and less curved cervical groove (more curved in *P. longipes*). These differences, added to the stratigraphic gap (Callovian-Kimmeridgian) separating both species, lead us to consider *P. nantosueltae* as a distinct species, albeit closely allied to *P. longipes*.

### General discussion

In extant lobsters, a long larval stage generally goes together with large brood (i.e., large number of smaller eggs per clutch), while species that hatch directly as adult-looking individuals generally produce a small number of relatively large eggs (Fig. 1). For instance, spiny and slipper lobsters (Achelata) produce in the range of tens of thousands to almost two million small eggs per clutch and live quite a long time as long-legged planktic larvae (phyllosoma; Table S1). In contrast, marine clawed lobsters and freshwater crayfishes (Astacidea) produce fewer (only a few hundred per clutch) but bigger eggs and display a shorter, more direct development^5^. Palaeontology and phylogenetics suggest that the ancestral state in decapod crustaceans is a relatively long and direct development pattern, without dramatic changes at each ontogenetic stage^8,9,21^.

Since the development of polychelidan lobsters incorporated a long-lived, giant planktic larval stage at the end of the Mesozoic^7^, it is surprising that their brood and egg sizes do not seem to have changed much in 165 Ma. Physiological or environmental constraints may have been at play. There is no data on the relationship between reproductive strategy and environmental conditions in extant polychelidan lobsters; however, the relationship between habitat and brood size has been reported in slipper lobsters. Species of slipper lobsters with a pelagic phyllosoma stage produce relatively small clutches of big eggs associated with a short-lived phyllosoma stage, while species with a pelagic phyllosoma stage produce very large clutches of small eggs associated with a longer-lived phyllosoma stage^22^. These specific behaviours are directly related to the larval survival rates in both environments, which is higher in coastal settings^22^, due to a lower dispersion and higher food availability in this environment compared to the open ocean.

The establishment of the long-lived planktic larva (eryoneicus), probably during the Cretaceous^7^ or after, likely offered the possibility to polychelidan lobsters to survive in the deep sea, while shallow water taxa went extinct^1^, possibly owing to the evolution of true crabs that likely competed for similar resources and habitats^23,24^. At the same time, the limited resources of this harsh environment may have been constraining brood size in polychelidan lobsters. Eryoneicus larvae develop higher in the water column than the deep sea, alleviating competition with their parents for food resources on the seafloor^25^. This giant larval stage thus likely increased the fitness of polychelidan lobsters and allowed their wide geographical dispersal^25^, despite a comparatively small brood size.

## Material and Methods

### Specimens

The fossil specimen is embedded within an unbroken nodule (MNHN.F.A58254, Fig. 3A,B) from the La Voulte-sur-Rhône Lagerstätte, Callovian, France, and is housed in the palaeontology collection of the Muséum national d’Histoire naturelle (acronym: MNHN.F). The La Voulte-sur-Rhône Lagerstätte is world renowned for the diversity and quality of its fauna, especially arthropods^19,26^. Fossiliferous concretions with exceptional preservation come from marls outcropping at the Ravin des Mines locality, which are topped by 15 m of iron carbonate deposits^19^.

Comparisons were made with extant specimens housed in the zoological collections of the Muséum national d’Histoire naturelle (acronym: MNHN-IU) and to fossil specimens of *Palaeopolycheles longipes* (Fraas, 1855; Fig. S4) housed in the Staatliches Museum für Naturkunde, Stuttgart (acronym: SMNS). The new database of egg size in modern polychelidan lobsters was generated for comparison from the MNHN collections. 10 egg diameters were measured per specimen (photograph) using ImageJ. Statistical difference between each extant specimen and MNHN.F.A58254 were tested using a Wilcoxon-Mann-Whitney test.

### Microtomography

For microtomography, we used the same method as described in Jauvion *et al*.^2^. The unbroken nodule was imaged with a v│tome│× 240 L tomograph (GE Sensing & Inspection Technologies Phoenix ×│ray) equipped with a microfocus 240 kV/320 W tube delivering a current/voltage of 220 mA/120 kV. Microtomography was performed at the AST-RX technical platform of the MNHN, Paris. Data were processed to obtain a series of virtual slices with a voxel size (cubic voxel) of 31.4 mm. Virtual slices were saved as a series of image files in 16 bits greyscale indicating differences in absorption of X-ray within the nodule (darker for low absorption, brighter for high absorption). 1900 virtual slices with a resolution of 1340 × 1198 pixels were thus obtained. Outlines of the fossilized structure were segmented using Mimics 20.0 (Materialise) for 3D reconstruction. MeshLab was used for 3D rendering.

## Supplementary information

Supplementary information.

## Data Availability

X-Ray microtomogaphy data (virtual slices) are available here^27^: 10.5281/zenodo.3624687.
