# Supplementary material for: A new polychelidan lobster preserved with its eggs in a 165 Ma nodule
Source: Sci Rep. 2020 Feb 27;10:3574. doi: 10.1038/s41598-020-60282-1 (PMC7046737; doi:10.1038/s41598-020-60282-1)
Supplement: Supplementary file 1 — Supplementary information. [file 41598_2020_60282_MOESM1_ESM.docx]

Supplementary Information - **A new polychelidan lobster preserved with its eggs in a 165 Ma nodule**

Clément Jauvion^1,2,*^, Denis Audo^3,4^, Sylvain Bernard^2^, Jean Vannier^5^, Allison C. Daley^6^ & Sylvain Charbonnier^1^

^1^Muséum national d'Histoire naturelle, Sorbonne Université, CNRS UMR 7207, CR2P, Centre de Recherche en Paléontologie - Paris, 8 rue Buffon, 75005 Paris (France); e-mails: [clement.jauvion@mnhn.fr](mailto:clement.jauvion@mnhn.fr); [sylvain.charbonnier@mnhn.fr](mailto:sylvain.charbonnier@mnhn.fr)

^2^Muséum national d'Histoire naturelle, Sorbonne Université, CNRS UMR 7590, IRD, Institut de Minéralogie, de Physique des Matériaux et de Cosmochimie, IMPMC, Paris (France); e-mail: [sylvain.bernard@mnhn.fr](mailto:sylvain.bernard@mnhn.fr)

^3^Yunnan Key Laboratory for Palaeobiology, Yunnan University, China

^4^MEC International Joint Laboratory for Palaeobiology and Palaeoenvironment, Yunnan University, China; e-mail: [daudo@edu.mnhn.fr](mailto:daudo@edu.mnhn.fr)

^5^Univ Lyon, Université Claude Bernard Lyon 1, ENS de Lyon, CNRS, UMR 5276 LGL-TPE, 2, rue Raphaël Dubois, 69622 Villeurbanne Cedex, France; e-mail: [jean.vannier@univ-lyon1.fr](mailto:jean.vannier@univ-lyon1.fr)

^6^Institute of Earth Sciences, University of Lausanne, Géopolis, CH-1015 Lausanne, Switzerland; e-mail: [allison.daley@unil.ch](mailto:allison.daley@unil.ch)

*Corresponding author

**List of cited taxa and associated references, in alphabetical order**

Achelata Scholtz and Richter, 1995^1^

*Astacus leptodactylus* Escholtz, 1823, accepted as *Pontastacus leptodactylus* ([Eschscholtz](https://fr.wikipedia.org/wiki/Johann_Friedrich_von_Eschscholtz), [1823](https://fr.wikipedia.org/wiki/1823))^2^

*Austropotamobius italicus* (Faxon, 1914)^3^*,* accepted as *Austropotamobius fulcisianus orientalis* (Karaman, 1929)^4^

*Austropotamobius torrentium* (von Paula Schrank, 1803)^5^

Astacidea Latreille, 1802^6^

Astacoidea Latreille, 1802^6^

*Cambaroides japonicus* (De Haan, 1814 [in De Haan, 1833-1850])^7^

*Eryon longipes* Fraas, 1855^8^

Eucrustacea Kingsley, 1894^9^

*Homarus* *gammarus* (Linnaeus, 1758)^10^

*Ibacus* Leach, 1815^11^

*Ibacus alticrenatus* Bate, 1888^12^

*Ibacus brucei* Holthuis, 1977^13^

*Ibacus chacei* Brown & Holthuis, 1998^14^

*Ibacus peronii* Leach, 1815^11^

*Jasus edwardsii* (Hutton, 1875)^15^

*Nephrops norvegicus* (Linnaeus, 1758)^10^

*Palaeopentacheles roettenbacheri* (Münster, 1839)^16^

*Palaeopolycheles* Knebel, 1907^17^

*Palaeopolycheles longipes* (Fraas, 1855)^8^

Palinuridae Latreille, 1802^6^

*Panulirus argus* (Latreille, 1804)^18^

*Panulirus homarus* (Linnaeus, 1758)^10^

*Panulirus marginatus* (Quoy & Gaimard, 1825)^19^

*Panulirus penicillatus* (Olivier, 1791)^20^

Pleocyemata Burkenroad, 1963^21^

*Polycheles coccifer* Galil, 2000^22^

*Polycheles enthrix* (Bate, 1878)^23^

*Polycheles typhlops* Heller, 1862^24^

*Pentacheles laevis* Bate, 1878^23^

Polychelida Scholtz and Richter, 1995^1^

Scyllaridae Latreille, 1825^25^

*Scyllarides* Gill, 1898^26^

*Scyllarides astori* Holthuis, 1960^27^

*Scyllarides deceptor* Holthuis, 1963^28^

*Scyllarides delfosi* Holthuis, 1960^27^

*Scyllarides latus* (Latreille, 1802)^6^

*Scyllarides squammosus* (H. Milne Edwards, 1837)^29^

*Stereomastis aculeata* (Galil, 2000)^22^

*Stereomastis auriculata* (Bate, 1878)^23^

*Stereomastis galil* (Ahyong & Brown, 2002)^30^

*Stereomastis helleri* (Bate, 1878)^23^

*Tethyseryon campanicus* Bravi, Garassino, Bartiromo, Audo, Charbonnier, Schweigert, Thévenard & Longobardi, 2014^31^

*Thenus orientalis* (Lund, 1793)^32^

**List of abbreviations:**

CSMNF – Museo di Paleontologia de l’Università degli Studi di Napoli “Federico II” (Naples, Italy)

MB.A – Museum für Naturkunde (Berlin, Germany)

MNHN.F – Palaeontological collections of the Muséum national d’Histoire naturelle (Paris, France)

SMNS – Staatliches Museum für Naturkunde (Stuttgart, Germany)


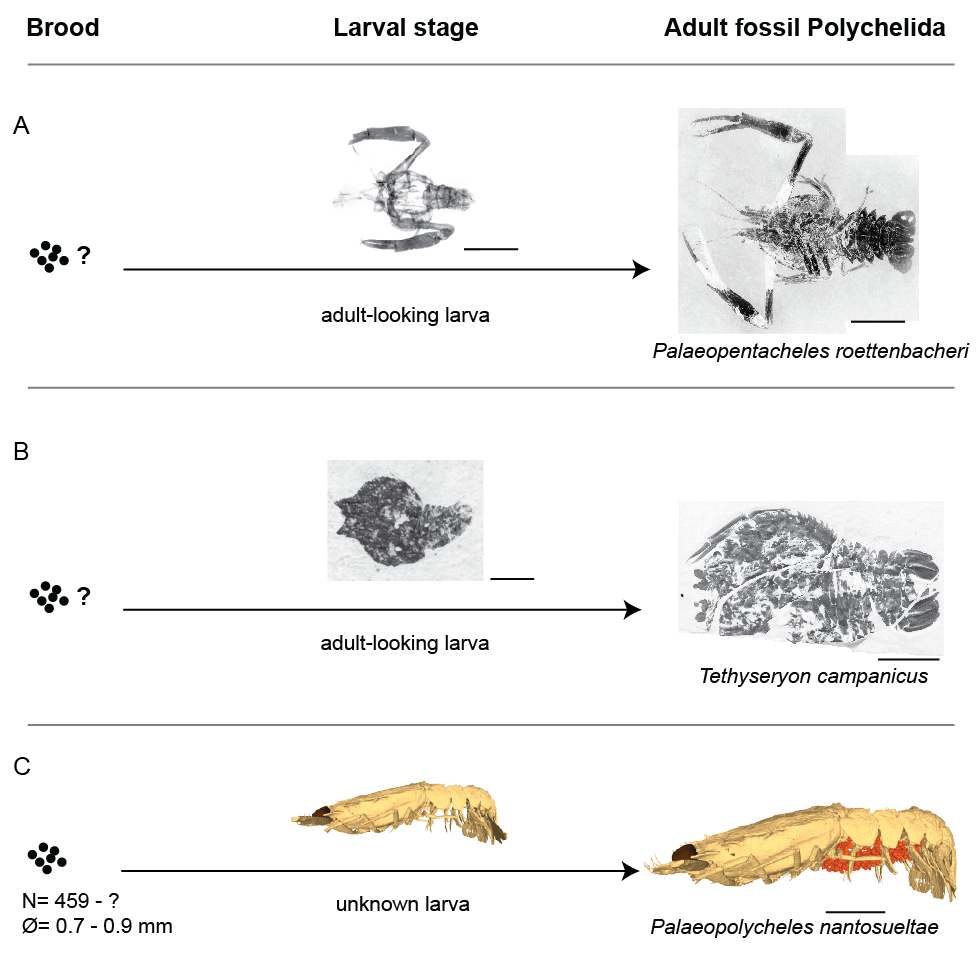


Fig. S1. Brood and development strategies in Jurassic polychelidan lobsters. A, *Palaeopentacheles roettenbacheri* (Late Jurassic, Germany^33^; pictures by S. Eiler and J.T. Haug), eggs, larval (SMNS 67903) and adult (MB.A 1118) stages; B, *Tethyseryon campanicus* (Middle Jurassic, Italy^31^), eggs, larval (CSMNF 22000e) and adult (CSMNF 22000a) stages (pictures by D. Audo); C, *Palaeopolycheles nantosueltae* (Middle Jurassic, France), eggs, larval and adult stages. Scale bars (from top left to bottom right): 5 mm, 20 mm, 2 mm, 10 mm and 10 mm.


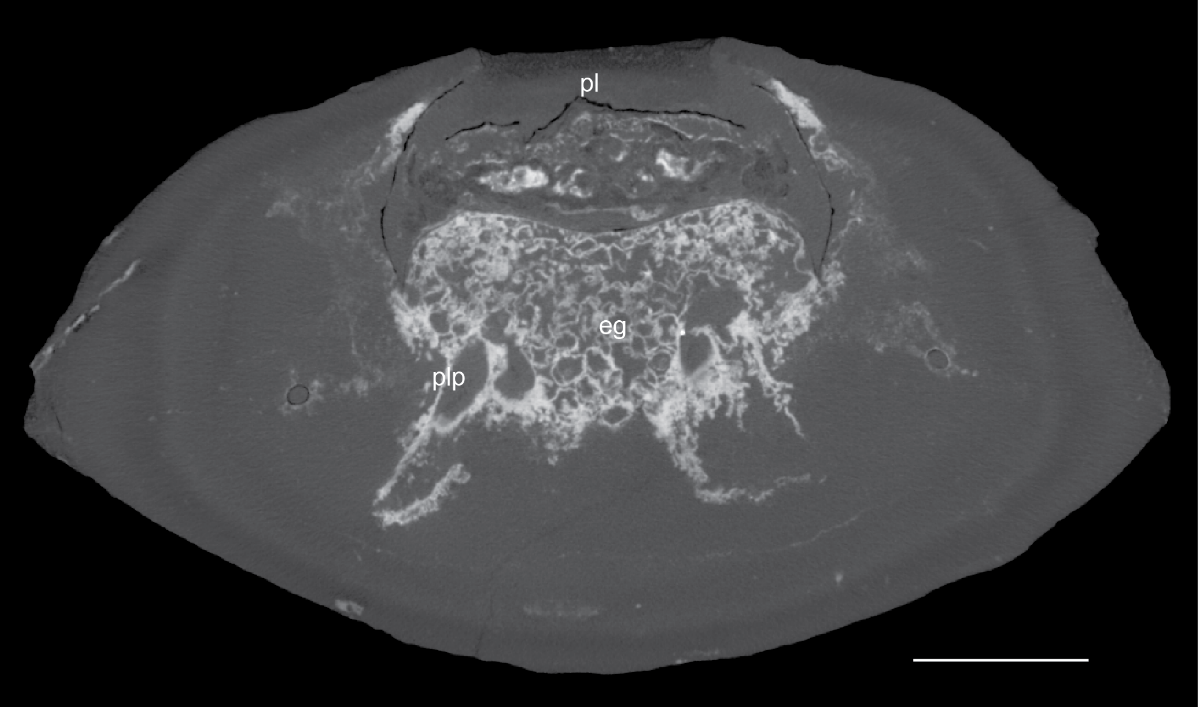


Fig. S2. Tomographic slide through MNHN.F.A58254 (cross section) displaying the preserved eggs under the pleon. eg, eggs; pl, pleon; plp, pleopod. Scale bar: 5 mm.


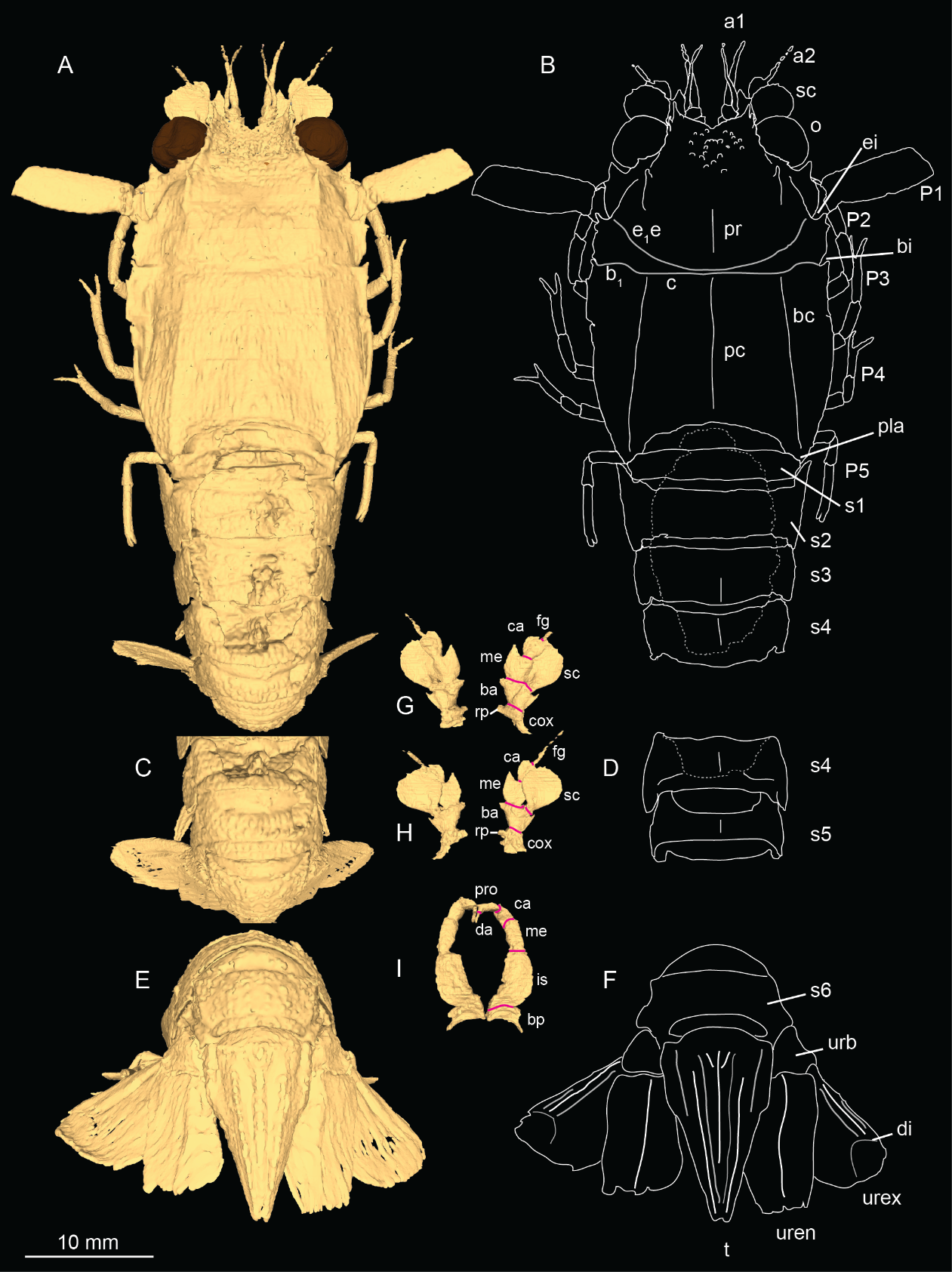
Fig. S3. Anatomical characteristics of *Palaeopolycheles nantosueltae*. A, C, E, dorsal views of the 3D model; B, D, F, corresponding interpretative line drawings; G-H, antennae (a2), 3D model, ventral (G) and dorsal views (H); I, third pair of maxillipeds (mxp3), ventral view of 3D model. a1, antennula ; a2, antenna; b_1_, hepatic groove; ba, basis; bc, branchial carina; bi, hepatic incision; bp, basal podomeres; c, postcervical groove; ca, carpus; cox, coxa; da, dactylus; di, diaeresis; e_1_e, cervical groove; ei, cervical incision; fg, flagellum; is, ischium; me, merus; o, eye; P1-5, pereiopods 1-5 (thoracopods 4-8); pc, postcervical carina; pla, posterolateral angle; pr, postrostral carina; pro, propodus; s1-6, pleonites 1-6; rp, renal process; sc, scaphocerite; t, telson; urb, uropodal basipod; uren, uropodal endopod; urex, uropodal exopod. Scale bar: 10 mm. Drawing by C. Jauvion.
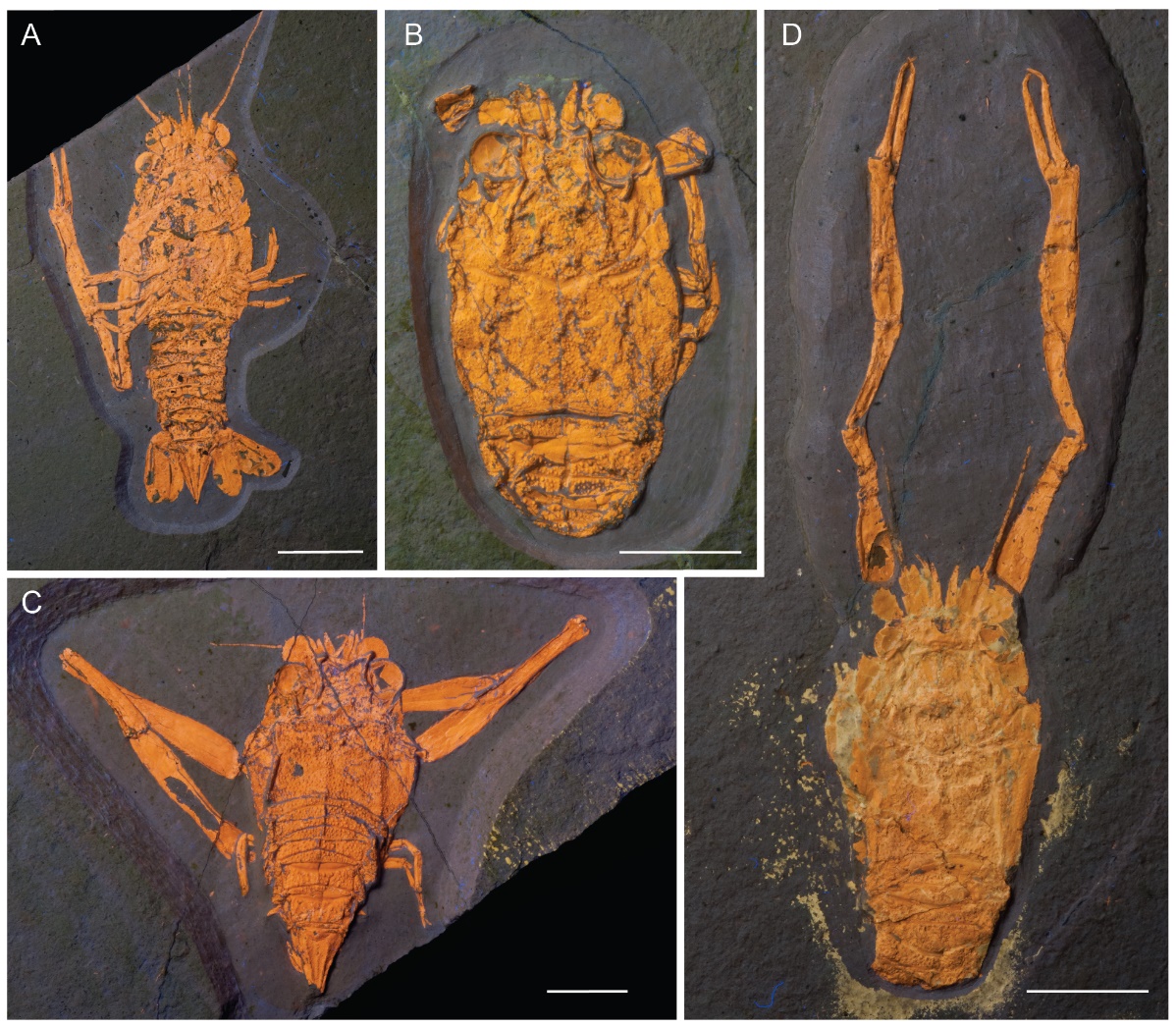


Fig. S4. *Palaeopolycheles longipes*. A, SMNS 63833; B, SMNS 63729; C, SMNS 63724; D, SMNS 70203a. Photographs by D. Audo using UV illumination associated with an orange filter. Scale bars: 10 mm.

Table S1. Comparative table of brood care parameters in extant achelate and clawed lobsters (incl. crayfishes).

| Group | Brood size (number of eggs) | Egg size (mm) | Large planktic larval stage | References |
| --- | --- | --- | --- | --- |
| Astacidea Latreille, 1802 |  |  | no |  |
| Astacoidea Latreille, 1802 |  |  | - |  |
| *Austropotamobius italicus* | 26 - 299 | - |  | Galeotti *et al.* 2006^34^ |
| *Austropotamobius torrentium* | 30 - 104 | 2.4 - 3.4 |  | Maguire *et al.* 2005^35^ |
| *Cambaroides japonicus* | 22 - 75 | 2.12 - 2.15 |  | Nakata & Goshima 2004^36^ |
| Nephropoidea Dana, 1852 |  |  | - |  |
| *Homarus gammarus* | 4,000 - 40,000 | 1.5 - 2.1 |  | Agnalt 2008^37^, original data |
| *Homarus gammarus* | 2,000 - 44,000 | - |  | Agnalt 2008^37^, review |
| *Homarus gammarus* | 2,000 - 44,000 | weight given |  | Tully *et al.* 2001^38^ |
| *Nephrops norvegicus* | 100 - 1,500 | 1.68 +/- 0.09 |  | Mori *et al.* 1998^39^ |
| *Nephrops norvegicus* | 50 - 1,550 | - |  | Farmer 1974^40^ |
| *Nephrops norvegicus* | 500 - 6,400 | 1.24 - 1.68 (1 - 2.5 mm^3^) |  | Mori *et al.* 2001^41^ |
| Achelata Scholtz & Richter, 1995 |  |  | *phyllosoma* |  |
| Palinuridae Latreille, 1802 |  |  |  |  |
| *Jasus edwardsii* | 45,000 - 466,000 | - |  | Linnane *et al.* 2008^42^ |
| *Panulirus argus* | 100,000 - 1,600,000 | - |  | Fonseca-Larios & Briones-Fourzán 1998^43^ |
| *Panulirus homarus* | 50,000 - 550,000 | weight given |  | Vijayakumaran *et al.* 2005^44^ |
| *Panulirus marginatus* | 100,000 - 600,000 | 0.6 - 0.8 |  | DeMartini *et al*. 2003^45^ |
| *Panulirus penicillatus* | 60,000 - 475,000 | - |  | Plaut 1993^46^ |
| Scyllaridae Latreille, 1825  Coastal species (phyllosoma) |  |  |  |  |
| *Ibacus alticrenatus* | 1,734 - 14,762 | 0.94 - 1.29 |  | Haddy *et al.* 2005^47^ |
| *Ibacus brucei* | 2,049 - 61,339 | 0.73 - 1.01 |  | Haddy *et al.* 2005^47^ |
| *Ibacus chacei* | 2,117 - 28,793 | 1.02 - 1.37 |  | Haddy *et al.* 2005^47^ |
| *Ibacus peronii* | 5,500 - 37,000 | 1.18 +/- 0.013 |  | Stewart & Kenelly 1997^48^ |
| *Thenus orientalis* | 12,467 | 1 - 1.204 |  | Hossain 1975^49^ |
| Pelagic species (phyllosoma) |  |  |  |  |
| *Scyllarides astori* | 87,000 - 360,000 | - |  | Hearn & Toral-Granda 2007^50^ |
| *Scyllarides deceptor* | 58,871 - 517,675 | 0.62 - 0.72 |  | Oliveira *et al.* 2008^51^ |
| *Scyllarides delfosi* | 24,710 - 190,060 | 0.60 - 0.64 |  | Lima et al. 2018^52^ |
| *Scyllarides latus* | 99,572 - 107,105 | - |  | Almog-Shtayer 1988 cited in Oliveira *et al.* 2008^51^ |
| *Scyllarides latus* | 151,000 - 356,000 | 0.604 - 0.755 |  | Martins 1985^53^ |
| *Scyllarides squammosus* | 53,807 - 227,489 | 0.61 - 0.77 |  | DeMartini & Williams 2001^54^ |

Table S2. Database of measured egg sizes in modern and fossil (MNHN.F.A58254) polychelidan lobsters.

| Specimen number | MNHN-IU-2018-4212 | MNHN-IU-2018-4212 | MNHN-IU-2018-4212 |
| --- | --- | --- | --- |
| *Species* | *Polycheles enthrix* | *Polycheles enthrix* | *Polycheles enthrix* |
| Egg diameter (mm) | 0.51 | 0.55 | 0.57 |
|  | 0.53 | 0.49 | 0.60 |
|  | 0.53 | 0.50 | 0.70 |
|  | 0.52 | 0.50 | 0.64 |
|  | 0.47 | 0.51 | 0.53 |
|  | 0.51 | 0.51 | 0.64 |
|  | 0.45 | 0.50 | 0.58 |
|  | 0.48 | 0.54 | 0.53 |
|  | 0.47 | 0.54 | 0.61 |
|  | 0.51 | 0.46 | 0.53 |
|  |  |  |  |
| Specimen number | MNHN-IU-2018-4204 | MNHN-IU-2018-4210 | MNHN-IU-2016-9909 |
| *Species* | *Stereomastis aculeata* | *Pentacheles laevis* | *Stereomastis aculeata* |
| Egg diameter (mm) | 0.61 | 0.69 | 0.68 |
|  | 0.66 | 0.56 | 0.63 |
|  | 0.67 | 0.71 | 0.67 |
|  | 0.66 | 0.56 | 0.64 |
|  | 0.61 | 0.59 | 0.64 |
|  | 0.60 | 0.67 | 0.58 |
|  | 0.61 | 0.52 | 0.55 |
|  | 0.61 | 0.56 | 0.55 |
|  | 0.66 | 0.54 | 0.59 |
|  | 0.63 | 0.56 | 0.54 |
|  |  |  |  |
| Specimen number | MNHN-IU-2016-9911 | MNHN-IU-2008-14869 | MNHN-IU-2018-4215 |
| *Species* | *Stereomastis galil* | *Stereomastis auriculata* | *Pentacheles laevis* |
| Egg diameter (mm) | 0.62 | 0.48 | 0.65 |
|  | 0.66 | 0.65 | 0.55 |
|  | 0.55 | 0.60 | 0.60 |
|  | 0.64 | 0.60 | 0.58 |
|  | 0.59 | 0.55 | 0.60 |
|  | 0.61 | 0.59 | 0.60 |
|  | 0.60 | 0.65 | 0.63 |
|  | 0.64 | 0.62 | 0.56 |
|  | 0.55 | 0.60 | 0.49 |
|  | 0.56 | 0.52 | 0.63 |
|  |  |  |  |
| Specimen number | MNHN-IU-2018-4215 | MNHN-IU-2016-9914 | MNHN-IU-2013-18792 |
| *Species* | *Pentacheles laevis* | *Stereomastis auriculata* | *Polycheles typhlops* |
| Egg diameter (mm) | 0.57 | 0.62 | 0.51 |
|  | 0.59 | 0.76 | 0.57 |
|  | 0.60 | 0.69 | 0.51 |
|  | 0.57 | 0.64 | 0.50 |
|  | 0.50 | 0.62 | 0.53 |
|  | 0.56 | 0.69 | 0.46 |
|  | 0.63 | 0.60 | 0.51 |
|  | 0.53 | 0.56 | 0.55 |
|  | 0.53 | 0.61 | 0.48 |
|  | 0.65 | 0.56 | 0.53 |
|  |  |  |  |
| Specimen number | MNHN-IU-2008-10470 | MNHN-IU-2018-4214 | MNHN-IU-2018-4211 |
| *Species* | *Stereomastis helleri* | *Polycheles coccifer* | *Polycheles typhlops* |
| Egg diameter (mm) | 0.83 | 0.53 | 0.44 |
|  | 0.82 | 0.53 | 0.45 |
|  | 0.83 | 0.51 | 0.50 |
|  | 0.84 | 0.46 | 0.52 |
|  | 0.79 | 0.48 | 0.45 |
|  | 0.79 | 0.54 | 0.43 |
|  | 0.77 | 0.47 | 0.45 |
|  | 0.86 | 0.50 | 0.47 |
|  | 0.77 | 0.51 | 0.47 |
|  | 0.84 | 0.53 | 0.48 |
|  |  |  |  |
| Specimen number | MNHN-IU-2018-4208 | MNHN-IU-2018-4209 | MNHN-IU-2018-4213 |
| *Species* | *Stereomastis aculeata* | *Polycheles enthrix* | *Stereomastis aculeata* |
| Egg diameter (mm) | 0.61 | 0.54 | 0.73 |
|  | 0.60 | 0.60 | 0.70 |
|  | 0.65 | 0.53 | 0.73 |
|  | 0.58 | 0.53 | 0.69 |
|  | 0.53 | 0.62 | 0.69 |
|  | 0.72 | 0.57 | 0.66 |
|  | 0.57 | 0.58 | 0.71 |
|  | 0.63 | 0.52 | 0.70 |
|  | 0.67 | 0.58 | 0.74 |
|  | 0.63 | 0.54 | 0.70 |
|  |  |  |  |
| Specimen number | MNHN-IU-2016-9924 | MNHN-IU-2018-4203 | MNHN-IU-2018-4203 |
| *Species* | *Stereomastis cerata* | *Stereomastis aculeata* | *Stereomastis aculeata* |
| Egg diameter (mm) | 0.73 | 0.60 | 0.58 |
|  | 0.73 | 0.66 | 0.62 |
|  | 0.80 | 0.62 | 0.62 |
|  | 0.69 | 0.62 | 0.64 |
|  | 0.75 | 0.62 | 0.58 |
|  | 0.77 | 0.66 | 0.59 |
|  | 0.73 | 0.74 | 0.64 |
|  | 0.75 | 0.69 | 0.62 |
|  | 0.73 | 0.65 | 0.57 |
|  | 0.73 | 0.70 | 0.59 |
|  |  |  |  |
| Specimen number | MNHN.F.A58254 |  |  |
| *Species* | *Palaeopolycheles nantosueltae* | |  |
| Egg diameter (mm) | 0.94 |  |  |
|  | 0.80 |  |  |
|  | 0.80 |  |  |
|  | 0.59 |  |  |
|  | 0.80 |  |  |
|  | 0.95 |  |  |
|  | 0.74 |  |  |
|  | 0.79 |  |  |
|  | 0.81 |  |  |
|  | 0.75 |  |  |

**References**

1. Scholtz, G. & Richter, S. Phylogenetic systematics of the reptantian Decapoda (Crustacea, Malacostraca). *Zool. J. Linn. Soc.* **113**, 289–328 (1995).

2. Eschscholtz, F. F. Descriptio novae Astacorum speciei Rossicae. *Mem. la Soc. Imp. des Nat. du Moscu* **1**, 6:109-110, (1823).

3. Faxon, W. Notes on the crayfishes. *Memories Museum Comp. Zoölogy Harvard Coll.* **40**, 351–427 (1914).

4. Karaman, S. Die Potamobiiden Jugoslaviens. *Glas. Zemalj. muzeja u Bosni i Hercegovini* **41**, 147–150 (1929).

5. Paula Schrank, F. von. Fauna boica : durchgedachte Geschichte der in Baiern einheimschen und zahmen Thiere. in (1803). doi:10.5962/bhl.title.51801.

6. Latreille, P. A. *Histoire naturelle, générale et particulière, des crustacés et des insectes*. vol. 3 (F. Dufart, 1802).

7. De Haan, W. Crustacea. in *In: von Siebold, P.F., Fauna Japonica sive Descriptio Animalium, quae in Itinere per Japoniam, Jussu et Auspiciis Superiorum, qui Summum in India Batava Imperium Tenent, Suspecto, Annis 1823-1830 Collegit, Notis, Observationibus et Adumbrationibus Illustr* (1814).

8. Fraas, O. Beiträge zum obersten weissen Jura in Schwaben. *Jahreshefte des Vereins fur Vaterl. Naturkd. Wurttemb.* **11**, 76–106 (1855).

9. Kingsley, J. S. The classification of the Arthropoda. *Am. Nat.* **28**, 118–135 (1894).

10. Linnaeus, C. Systema Naturae per regna tria naturae, secundum classes, ordines, genera, species, cum characteribus, differentiis, synonymis, locis. *Ed. decima, reformata. Laurentius Salvius Holmiae* **824**, (1758).

11. Leach, W. E., Nodder, F. P., Nodder, R. P. & Shaw George. *The zoological miscellany : being descriptions of new, or interesting animals /*. vol. v.2 (1815) (London :Printed by B. McMillan for E. Nodder & Son and sold by all booksellers, 1815).

12. Bate, C. S. Report on the Crustacea Macrura collected by the Challenger during the years 1873-76. *Rep. Sci. Results Voyag. H.M.S. ”Challenger” Dur. years 1873-76* (1888).

13. Holthuis, L. B. Two new species od scyllarid lobsters (Crustacea Decapoda, Palinuridea) from Australia and the Kermadec Islands, New Zealand. *Zool. Meded.* **52**, 191–200 (1977).

14. Brown, D. E. & Holthuis, L. B. The Australian species of the genus Ibacus (Crustacea: Decapoda: Scyllaridae), with the description of a new species and addition of new records. *Zool. Meded.* **72**, 113–141 (1998).

15. Hutton, F. W. Description of two new Species of Crustacea from New Zealand. *Trans. Proc. R. Soc. New Zeal.* **7**, 279–280 (1874).

16. Münster, G. G. zu. Decapoda Macroura.Abbildung und Beschreibung der fossilen langschwänzigen Krebse in den Lakschiefern von Bayern. *Beiträge zur Petrefacten-kd.* **1**, 1–88 (1839).

17. von Knebel, W. Die Eryoniden des oberen Weissen Jura. *Arch. fur Biontologie* **2**, 195–233 (1907).

18. Latreille, P. A. *Histoire naturelle, générale et particulière des crustacés et des insectes: ouvrage faisant suite aux oeuvres de Leclerc de Buffon, et partie du cours complet d’histoire naturelle rédigé par CS Sonnini, membre de plusieurs Sociétés savantes*. vol. 13 (Dufart, 1804).

19. Quoy, J. R. C. & Gaimard, P. Section Ière. Des Crustacés. in *Voyage autour du Monde, Entrepris par Ordre du Roi,... éxécuté sur les corvettes de S. M. l’Uranie et la Physicienne, pendant les années 1817, 1818, 1819 et 1820. Zoologie.* (ed. de Freycinet, L.) 517–541 (Chez Pillet-Aîné, 1825).

20. Olivier, A. G. Histoire Naturelle. Insectes. Par M. Olivier. in *In: Encyclopédie Méthodique, ou par ordre de matières; par une société de gens de lettres de savans et d’artistes. Paris* (1791).

21. Burkenroad, M. D. The evolution of the Eucarida, (Crustacea, Eumalacostraca), in relation to the fossil record. *Tulane Stud. Geol.* **2**, 3–17 (1963).

22. Galil, B. S. Crustacea Decapoda: review of the genera and species of the family Polychelidae Wood-Mason, 1874. in *In: Crosnier, A. (Ed.) (2000). Results of the MUSORSTOM expeditions: 21. Mémoires du Muséum National d’Histoire Naturelle (Paris), A (Zoologie)* vol. 184 285–387 (2000).

23. Bate, C. S. XXXII.—On the Willemoesia group of Crustacea. *J. Nat. Hist.* **2**, 273–283 (1878).

24. Heller, C. Neue Crustaceen, gesammelt während der Weltumseglung der k.k. Fregatte Novara. Zweiter vorläufiger Bericht. *Verhandlungen der Kais. Zool. Gesellschaft Wien* **12**, 519–528 (1862).

25. Latreille, P. A. *Familles naturelles du règne animal*. (Baillière, 1825).

26. Gill, T. The crustacean genus Scyllarides. *Science (80-. ).* **7**, 98–99 (1898).

27. Holthuis, L. B. *Preliminary descriptions of one new genus, twelve new species and three new subspecies of scyllarid lobsters (Crustacea Decapoda Macrura)*. (1960).

28. Holthuis, L. B. Preliminary descriptions of some new species of Palinuridea (Crustacea, Decapoda, Macrura, Reptantia). *Proc. K. Ned. Akad. Wet. C.* **66**, 54–60 (1963).

29. Milne Edwards, H. *Histoire naturelle des crustacés : comprenant l’anatomie, la physiologie et la classification de ces animaux.* vol. t.2 (Paris :Librairie encyclopédique de Roret, 1837).

30. Ahyong, S. T. & Brown, D. E. New species and new records of Polychelidae from Australia (Crustacea: Decapoda). *Raffles Bull. Zool.* **50**, 53–80 (2002).

31. Bravi, S. *et al.* Middle Jurassic Monte Fallano Plattenkalk (Campania, southern Italy): first report on terrestrial plants, decapod crustaceans and fishes. *Neues Jahrb. für Geol. und Paläontologie - Abhandlungen* **272**, 79–107 (2014).

32. Lund, N. T. Slaegten Scyllarus. Jagttagelser til Insekternes Historie.I. *K. Danske Vidensk. Selsk. Skr.* **2**, 17–22 (1793).

33. Eiler, S. M. & Haug, J. T. Larval development of fossil polychelidan crustaceans, exemplified by the 150 million years old species *Palaeopentacheles roettenbacheri*. *Neues Jahrb. für Geol. und Paläontologie - Abhandlungen* **279**, 295–310 (2016).

34. Galeotti, P. *et al.* Female freshwater crayfish adjust egg and clutch size in relation to multiple male traits. *Proc. R. Soc. B Biol. Sci.* **273**, 1105–1110 (2006).

35. Maguire, I., Klobučar, G. I. V & Erben, R. The relationship between female size and egg size in the freshwater crayfish Austropotamobius torrentium. *Bull. Fr. Pêche Piscic.* **376**–**377**, 777–785 (2005).

36. Nakata, K. & Goshima, S. Fecundity of the Japanese crayfish, Cambaroides japonicus: ovary formation, egg number and egg size. *Aquaculture* **242**, 335–343 (2004).

37. Agnalt, A.-L. Fecundity of the European lobster (Homarus gammarus) off southwestern Norway after stock enhancement: do cultured females produce as many eggs as wild females? *ICES J. Mar. Sci.* **65**, 164–170 (2008).

38. Tully, O., Roantree, V. & Robinson, M. Maturity, fecundity and reproductive potential of the European lobster (Homarus gammarus) in Ireland. *J. Mar. Biol. Assoc. United Kingdom* **81**, 61–68 (2001).

39. Mori, M., Biagi, F. & De Ranieri, S. Fecundity and egg loss during incubation in Norway lobster (Nephrops norvegicus) in the North Tyrrhenian Sea. *J. Nat. Hist.* **32**, 1641–1650 (1998).

40. Farmer, A. S. D. Reproduction in Nephrops norvegicus (Decapoda: Nephropidae). *J. Zool.* **174**, 161–183 (1974).

41. Mori, M., Modena, M. & Biagi, F. Fecundity and egg volume in Norway lobster (Nephrops norvegicus) from different depths in the northern Tyrrhenian Sea. *Sci. Mar.* **65**, 111–116 (2001).

42. Linnane, A. J., Penny, S. S. & Ward, T. M. Contrasting fecundity, size at maturity and reproductive potential of southern rock lobster Jasus edwardsii in two South Australian fishing regions. *J. Mar. Biol. Assoc. United Kingdom* **88**, 583–589 (2008).

43. Fonseca-Larios, M. E. & Briones-Fourzán, P. Fecundity of the spiny lobster Panulirus argus (Latreille, 1804) in the Caribbean coast of Mexico. *Bull. Mar. Sci.* **63**, 21–32 (1998).

44. Vijayakumaran, M. *et al.* Captive breeding of the spiny lobster, Panulirus homarus. *New Zeal. J. Mar. Freshw. Res.* **39**, 325–334 (2005).

45. DeMartini, E. E., DiNardo, G. T. & Williams, H. A. Temporal changes in population density, fecundity, and egg size of the Hawaiian spiny lobster (Panulirus marginatus) at Necker Bank, Northwestern Hawaiian Islands. *Fish. Bull.* **101**, 22–31 (2003).

46. Plaut, I. Sexual Maturity, Reproductive Season and Fecundity of the Spiny Lobster Panulirus penicillatus from the Gulf of Eilat (Aqaba), Red Sea. *Aust. J. Mar. Freshw. Res.* **44**, 527–35 (1993).

47. Haddy, J. A., Courtney, A. J. & Roy, D. P. ASPECTS OF THE REPRODUCTIVE BIOLOGY AND GROWTH OF BALMAIN BUGS (IBACUS SPP.) (SCYLLARIDAE). *J. Crustac. Biol.* **25**, 263–273 (2005).

48. Stewart, J. & Kennelly, S. J. Fecundity and Egg-Size of the Balmain Bug Ibacus peronii (Leach, 1815) (Decapoda, Scyllaridae) off the East Coast of Australia. *Crustaceana* **70**, 191–197 (1997).

49. Hossain, M. A. On the squat lobster, Thenus orientalis (Lund) off Visakhapatnam (Bay of Bengal). *Curr. Sci.* **44**, 161–162 (1975).

50. Hearn, A. & Toral-Granda, M. V. Reproductive biology of the red spiny lobster, Panulirus penicillatus and the Galapagos slipper lobster, Scyllarides astori in the Galapagos Islands. *Crustaceana* **80**, 297–312 (2007).

51. Oliveira, G., Freire, A. S. & Bertuol, P. R. K. Reproductive biology of the slipper lobster Scyllarides deceptor (Decapoda: Scyllaridae) along the southern Brazilian coast. *J. Mar. Biol. Assoc. United Kingdom* **88**, 1433–1440 (2008).

52. Lima, F. A., Martinelli-Lemos, J. M., Silva, K. C. A., Klautau, A. G. M. & Cintra, I. H. A. Population structure and fecundity of Scyllarides delfosi Holthuis, 1960 (Scyllaridae) on the Amazon continental shelf. *Crustaceana* **91**, 1027–1037 (2018).

53. Martins, H. R. Biological Studies of the Exploited Stock of the Mediterranean Locust Lobster Scyllarides latus (Latreille, 1803) (Decapoda: Scyllaridae) in the Azores. *J. Crustac. Biol.* **5**, 294–305 (1985).

54. DeMartini, E. E. & Williams, H. A. Fecundity and egg size of Scyllarides squammosus (Decapoda: Scyllaridae) at Maro Reef, Northwester Hawaiian Islands. *J. Crustac. Biol.* **21**, 891–896 (2001).
